# Prospective BMI changes in preschool children are associated with parental characteristics and body weight perceptions: the ToyBox-study

**DOI:** 10.1017/S1368980021001518

**Published:** 2022-06

**Authors:** Yannis Manios, Katrina A Lambert, Eva Karaglani, Christina Mavrogianni, Luis A Moreno Aznar, Violeta Iotova, Anna Świąder-Leśniak, Berthold Koletzko, Greet Cardon, Odysseas Androutsos, George Moschonis, Toy Box Study Group

**Affiliations:** 1 Department of Nutrition and Dietetics, School of Health Science and Education, Harokopio University, 70 El Venizelou Avenue, Kallithea 17671, Athens, Greece; 2 Department of Public Health, School of Psychology and Public Health, La Trobe University, Melbourne, Victoria, Australia; 3 Department of Nutrition and Dietetics, School of Physical Education, Sport Science and Dietetics, University of Thessaly, Volos, Greece; 4 GENUD (Growth, Exercise, NUtrition and Development) Research Group, University of Zaragoza, C/Corona de Aragon, Zaragoza, Spain; 5 School of Health Science (EUCS), University of Zaragoza, C/Domingo Miral s/n, Zaragoza, Spain; 6 Department of Pediatrics, Medical University Varna, Varna, Bulgaria; 7 The Children’s Memorial Health Institute, Warsaw, Poland; 8 Ludwig-Maximilians-Universität Munich, Dr von Hauner Children’s Hospital, University of Munich Medical Centre, Munich, Germany; 9 Department of Movement and Sports Sciences, Ghent University, Watersportlaan 2, Ghent, Belgium; 10 Department of Dietetics, Nutrition and Sport, School of Allied Health, Human Services and Sport, La Trobe University, Melbourne, Victoria, Australia

**Keywords:** Preschool, Lifestyle, Perinatal

## Abstract

**Objective:**

To examine the effect of the intervention implemented in the ToyBox-study on changes observed in age- and sex-specific BMI percentile and investigate the role of perinatal factors, parental perceptions and characteristics on this change.

**Design:**

A multicomponent, kindergarten-based, family-involved intervention with a cluster-randomised design. A standardised protocol was used to measure children’s body weight and height. Information was also collected from parents/caregivers via the use of validated questionnaires. Linear mixed effect models with random intercept for country, socio-economic status and school were used.

**Setting:**

Selected preschools within the provinces of Oost-Flanders and West-Flanders (Belgium), Varna (Bulgaria), Bavaria (Germany), Attica (Greece), Mazowieckie (Poland) and Zaragoza (Spain).

**Participants:**

A sample of 6268 preschoolers aged 3·5–5·5 years (51·9 % boys).

**Results:**

There was no intervention effect on the change in children’s BMI percentile. However, parents’ underestimation of their children’s actual weight status, parental overweight and mothers’ pre-pregnancy overweight/obesity were found to be significantly and independently associated with increases in children’s BMI percentile in multivariate modelling.

**Conclusions:**

As part of a wide public health initiative or as part of a counseling intervention programme, it is important to assist parents/caregivers to correctly perceive their own and their children’s weight status. Recognition of excessive weight by parents/caregivers can increase their readiness to change and as such facilitate higher adherence to favourable behavioural changes within the family.

The increased prevalence of childhood obesity and the associated co-morbidities is a major public health concern. Approximately one-third of obese and overweight children continue to follow a higher BMI trajectory throughout the life course^(1)^. It is also well established that adults, who were obese as children, have an increased risk of morbidity and mortality, independent of their current body weight^(2,3)^.

Behaviours which lead to a positive energy balance and consequently to a gradual increase in body weight are established early in life. Consequently, preschool age is a key period for the implementation of behavioural change intervention. Energy balance-related behaviours (EBRB) in preschool years, such as limited engagement into physical activities and increased engagement into sedentary activities (i.e. TV viewing, playing video games etc.), have been associated with obesity in preschool children^(4,5)^. In preschool children, the evidence on relevant associations between obesity and dietary behaviour is also strong but mixed in terms of different dietary behaviours reported to be significantly associated with obesity by different studies^(5)^. However, despite the existing evidence on significant associations between EBRB and childhood obesity in preschool years, the effect of behavioural change interventions that attempt to positively modify EBRB in this young age group is still not adequately explored.

Preschool children do not entirely regulate their own EBRB, as they considerably rely on their parents/guardians. In this context, parental perception of their child’s body weight seems to play a significant role on their children’s EBRB and weight trajectory. More than half of overweight/obese children have their weight status underestimated by their parents^(6)^. Parents’ underestimation of their child’s increased body weight could be a significant barrier to the modification of ERBR^(7)^, since parents who do not recognise their children’s overweight status will probably not take any corrective actions. In addition, parental characteristics can have a significant effect on parental modelling, which can subsequently influence children’s EBRB^(8)^. In this regard, parental overweight and obesity produces and sustains an obesogenic environment at home that has been associated with obesity in preschool children in several countries worldwide^(9–11)^ as well as with rapid increases in BMI percentile during preschool years^(12)^.

Further to the effect of social environmental factors on EBRB and weight status of preschool children, a plethora of evidence also highlights the significant effect of factors occurring during early life stages on the subsequent development and growth of children^(13,14)^. There is much to support the linkage of perinatal factors with overweight and obesity in preschool children. As such, maternal pre-pregnancy weight has been associated with a significant risk of childhood overweight and obesity at ages 3, 4 and 5 years^(15–20)^. A systematic review and meta-analysis considering both preschool and elementary age children also reported that pre-pregnancy overweight was associated with two- and threefold increased likelihood of childhood overweight/obesity, respectively^(21)^. Furthermore, gestational weight gain above the recommended threshold has been independently associated with risks of overweight/obesity in early childhood^(22,23)^, although the effect relative to that of maternal pre-pregnancy overweight has been reported to be smaller^(23)^. In addition, maternal smoking during pregnancy has been associated with a higher BMI in preschool-aged children^(24,25)^, with a systematic review and meta-analysis suggesting an increased risk of overweight in childhood of 50 %^(25)^. Maternal parity has also been associated with a lower risk of childhood overweight^(26)^, although this maybe dependent on breast-feeding^(27)^. Exclusive breast-feeding and later age of introduction of non-breast milk have shown protective effects on preschool overweight/obesity in some^(28–30)^ but not all studies. Despite the well-established relationship between perinatal and early life factors with overweight and obesity at preschool years, their effect on BMI changes in this age group remains to be thoroughly investigated.

The ToyBox-study designed, implemented and evaluated a kindergarten-based, family-involved intervention to 3·5–5·5-year-old children from six European countries, who were followed-up for 1 year. The ToyBox-intervention focused on four different EBRB (water consumption, healthy snacking, sedentary behaviour and physical activity) and has successfully demonstrated decreases in prepacked fruit juice consumption^(31)^ and computer/video games use^(32)^.

This study aimed to investigate whether the modification of EBRB attributed to the Toybox intervention could potentially have a positive effect on the changes observed on children’s age- and sex-specific BMI percentile. By also recording a wide range of other potentially determining factors, the secondary aim of the present study was to assess the effect of perinatal factors and parental perceptions, socio-demographic and anthropometric characteristics on children’s BMI percentile change.

## Methods

### ToyBox-study

The ToyBox-intervention study was a multicomponent, kindergarten-based, family-involved intervention with a cluster-randomised design. The intervention focused on modifying EBRB (see online supplementary material, Supplementary Table 1). A detailed description of the design of the ToyBox-study is provided elsewhere^(33)^.

In brief, a standardised, multistage sampling approach was applied on preschool children aged 3·5–5·5 years old and their families, who were recruited from randomly selected municipalities within the provinces of Oost-Flanders and West-Flanders (Belgium), Varna (Bulgaria), Bavaria (Germany), Attica (Greece), Mazowieckie (Poland) and Zaragoza (Spain) (see online supplementary material, Supplementary Table 2). Randomisation of the recruited municipalities to intervention and control groups was conducted centrally by the coordinating centre, after the completion of baseline measurements. The municipalities were assigned to the intervention or control group in a 2:1 ratio within each of the three socio-economic status groups. Since the randomisation was conducted at a municipality level, the 309 kindergartens within each municipality were automatically allocated to the intervention or control group. Kindergartens were randomly selected within each municipality.

Intervention mapping protocol was used to plan and develop the standardised ToyBox and provides a systematic, stepwise framework for planning, implementing and evaluating an intervention based on existing scientific literature, theories and research data. Seven hundred kindergarten teachers were trained and delivered the programme in the kindergartens. For evaluation purposes, data were obtained both at baseline and follow-up from more than 5500 parents, indicating significant improvements on the targeted obesogenic behaviours among children and parents, as well as relevant parental practices. The intervention ran for 24 weeks and consisted of four modules on water consumption, healthy snacking, sedentary behaviour and physical activity delivered by the kindergarten teacher. Each model had a four-week ‘first focus’ and a two-week ‘repetition’ phase^(33)^.

### Data collection

Data were collected from 7541 preschool children and their parents/caregivers from May to June 2012 and 2013. Children’s anthropometrics were measured at baseline and 1-year post intervention, while parents/caregivers self-reported sociodemographic, lifestyle and perinatal information in questionnaires, administered at baseline and the 1-year follow-up.

### Energy balance-related behaviour measurement

Both in the baseline and follow-up measurements, parents/caregivers were asked to describe the child’s usual food and beverage habits over the last 12 months in a FFQ for young children. Items regarding beverage consumption included plain water, soft drinks, light soft drinks, home-made and freshly squeezed fruit juice, pre-packed/bottled fruit juice, tea, smoothies (all kinds), plain milk and sugared or chocolate milk.

Snacking behaviours were also measured using the FFQ completed by the child’s guardian, with snacks defined as a small portion of food eaten in between regular meals. Intakes of yogurt (i.e. plain yogurt), sugared or aromatised yogurt, cheese, fresh fruit, raw vegetables, milk-based desserts (e.g. chocolate mousse, ice cream, custard), chocolate and candy bars (e.g. plain chocolate bars, chocolate candy bars), sugar-based desserts (e.g. hard candies, jelly beans, lollipops), cakes, biscuits and salty snacks (e.g. potato chips) were each assessed.

Questions regarding sedentary behaviour were answered by parents/guardians as part of the Primary Caregivers’ Questionnaire. The amount of time that children spent on TV/DVD/video viewing, computer/video games use and quiet play on weekdays and weekend days was assessed in three questions of the Primary Caregivers’ Questionnaire (one for each SB subcomponent, i.e. TV viewing, computer/video games use and quiet play). For example, ‘How much time does your child spend on TV-viewing (a) on weekdays, and (b) on weekend days?’. Possible answer options varied on a nine-point Likert-type scale, ranging from ‘never’ to ‘more than 8 hours/day’.

Physical activity was measured objectively by step counts. Before the start of the intervention, baseline measurements were performed on weekdays from May to June 2012. On those days, researchers visited intervention and control kindergartens and fitted those preschool children with an accelerometer (Belgium) or a pedometer (other countries). One year later, from May to June 2013, follow-up measurements were performed.

### Anthropometric measures

Researchers in all participating centres were rigorously trained to conduct anthropometric measurements following a standardised procedure^(34,35)^. More specifically, researchers conducted two consecutive measurements of children’s body weight to the nearest 100 g and of children’s height, to the nearest 1 mm, using the same types of electronic scales (SECA 861 and SECA 813; Seca) and height using portable stadiometers (types SECA 225 and SECA 214; Seca), respectively. If the difference between first and second measurement was ≥1 kg in the case of weight, and ≥2 cm in the case of height, a third measurement was required and the two closest measurements were averaged.

Baseline and follow-up BMI percentile and *z*-scores were calculated for each child using the WHO ANTHRO software for those less than or equal to 60 months, and the WHO ANTHRO-PLUS software for those over 60 months. Children were categorised as: ‘underweight’, ‘normal weight’, ‘overweight’ or ‘obese’, based on the appropriate *z*-score. BMI percentile change in 1 year was defined as the difference of age- and sex-specific BMI percentile from baseline to 1-year post intervention (BMI percentile post intervention minus baseline BMI percentile) and was considered as the primary response variable in this analysis.

### Parental perception on children’s body weight

Parents/caregivers of children were also surveyed concerning their opinion about their child’s weight. Cross tabulation between BMI at baseline (per cut-off) and perception of child’s weight (‘What do you think about your child’s weight?’; response on a five-point scale) was used to evaluate the guardian’s ability to correctly identify their child’s body weight status at baseline. Correct perception was considered for: a child grouped as overweight/obese with parents’/caregivers’ response as ‘high’ or ‘very high’ child’s weight OR a child grouped as normal weight/underweight with response as ‘very low’ or ‘low’ or ‘normal’ child’s weight. Misperception was considered for: a child grouped as overweight/obese with response as very low/low/normal child’s weight.

### Parental socio-demographic and anthropometric characteristics

Children were categorised as ‘native’ if both biological parents were born in the country where the questionnaires were administered, whereas all other children as ‘non-native’ (i.e. having at least one parent born abroad). Parental body weight and height were self-reported by the parents/caregivers, and their BMI was calculated according to the Quetelet’s equation (weight (kg)/height^2^ (m^2^)). Parents/caregivers were categorised according to their BMI as ‘normal weight’ (≤24·9 kg/m^2^), ‘overweight’ (≥25 and ≤29·9 kg/m^2^) or ‘obese’ (≥30 kg/m^2^). Last, parents/caregivers were asked to report the level of their education.

### Perinatal and early life factors

Mothers were also asked to recall smoking habits during pregnancy (yes/no), pre-pregnancy weight, weight gained during pregnancy based on the classification recommended by the Institute of Medicine^(36)^, multiparous gestation, parity, birth weight and gestational age for the classification into ‘small for gestational age’ (<10th percentile), ‘appropriate for gestational age’ (10th–89th percentile) and ‘large for gestational age’ (⩾90th percentile), child’s feeding patterns from birth to 6 months of age (exclusive breast-feeding, exclusive formula or mixed), and child’s weight and length. Parents/guardians were asked to consult their child’s recorded infant’s growth chart/medical record and report the weight and length of their child at birth and at the 6th, 7th, 11th and 12th months of their child’s life. Using this information, change in weight-for-length *Z*-score from birth to 6 months of age was calculated and then classified into ‘poor’ (<−1 *Z*-score), ‘average’ (−1 to +1 *Z*-score) and ‘rapid’ (>+1 *Z*-score) weight gain during infancy.

### Statistical analysis

Differences between the control and intervention groups in descriptive variables were calculated by the *χ* ^2^ test. To account for the nested and hierarchical nature of the sampling linear mixed effect models were used to assess associations between BMI percentile change in 1 year and (i) intervention and (ii) perinatal factors, parental socio-demographic and anthropometric characteristics. Random intercepts were used for country, socio-economic status and kindergarten, with fixed mean for each country and the intercept varying among socio-economic status and kindergarten within socio-economic status.

A multivariate model was produced, containing the intervention effect and all factors found in univariate analysis to be significantly (*P* < 0·05) associated with change in BMI percentile, which were then eliminated from the multivariate model if not significant. Factors with potential collinearity issues were assessed separately and the strongest variable retain in the final model. We also tested for an interaction between the intervention and those factors that were found to be significantly (*P* < 0·05) associated with BMI percentile changes, in order to assess whether these factors could influence the effect of the intervention.

Regression coefficients are presented as beta with 95 % CI. Statistical analysis was performed in R statistical programme (version 3.5.3 (2019-03-11)). The level of significance was set as *P* < 0·05.

## Results

Table 1 presents the descriptive characteristics of the study sample. In total, 6268 children (51·9 % boys, mean age at baseline 4·74 ± 0·44 years) provided valid height and body weight data at baseline and follow-up. Previous attrition analyses^(32)^ showed no significant differences in age, sex and group of preschoolers who were lost to follow-up and those who were included (95 % CIage = 0·93, 1·16; 95 % CIsex = 0·92, 1·14; 95 % CIgroup = 0·91, 1·52). At baseline, the majority of children were under/normal weight (89·4 %), with 7·8 % overweight and 2·8 % obese. The mean change in BMI percentile was –1·48 (sd 13·49), with 907 children (14·5 %) increasing their BMI percentile by more than 10 %. The weight status of overweight/obese children was underestimated by 70·6 % of their parents or primary guardians (470/666 overweight/obese children; 7·5 % of total sample), and parents/guardians were more likely to underestimate the body weight of male children (*P* < 0·001). Conversely, only 2·3 % of parents/guardians overestimated the weight of their child. Just below 70 % of families had at least one parent/guardian who was overweight/obese.

**Table 1:** Socio-demographic, parental characteristics and perinatal factors in preschool children from six European countries (*n* 6268)

	Total sample (*n* 6268)		Control (*n* 2271)		Intervention (*n* 3997)		*P*-value^*^
	*n*	%	*n*	%	*n*	%	
Gender							0·982
Female	3011	48·0	1090	48·0	1921	48·1	
Male	3257	52·0	1181	52·0	2076	51·9	
European country							< 0·001
Belgium	1203	19·2	498	21·7	705	17·6	
Bulgaria	879	14·0	256	11·3	623	15·6	
Germany	988	15·8	384	16·9	604	15·1	
Greece	1012	16·1	285	12·5	727	18·2	
Poland	1320	21·1	536	23·6	784	19·6	
Spain	866	13·8	312	13·7	554	13·9	
Parents’/guardian’ perception of child’s weight status							0·856
Correct/over	5797	92·5	2103	92·6	3694	92·4	
Underestimation	470	7·5	168	7·4	302	7·6	
Children’s weight status at baseline							0·227
Underweight/normal	5601	89·4	2038	89·7	3563	89·2	
Overweight	490	7·8	180	7·9	310	7·8	
Obese	176	2·8	53	2·3	123	3·1	
Nationality							0·076
Native	5617	90·0	2056	91·7	3561	90·3	
Non-native parent/s	568	9·6	186	8·3	382	9·7	
Parental weight status							0·593
No parent overweight/obese	1533	30·1	578	31·0	955	29·6	
One parent overweight/obese	2616	51·4	945	50·6	1671	51·9	
Both parents overweight/obese	938	18·4	343	18·4	595	18·5	
Smoking during pregnancy							0·814
No smoking	4721	78·2	1723	78·4	2998	78·1	
Smoking	1316	21·8	475	21·6	841	21·9	
Gestational weight gain^**^							0·265
Meet IOM recommended weight gain	3507	62·7	1259	61·7	2248	63·3	
Exceed IOM recommended weight gain	2087	37·3	781	38·3	1306	36·7	
Gestational age							0·025
Full-term (≥37 week)	5720	91·3	2097	92·3	3623	90·6	
Pre-term (<37 weeks)	548	8·7	174	7·7	374	9·4	
Multiple birth							0·035
Single gestation	5606	93·3	2073	94·2	3533	92·7	
Multiple gestation	405	6·7	128	5·8	277	7·3	
Birth weight							0·061
Small for gestational age	598	10·4	192	9·2	406	11·1	
Normal for gestational age	4618	80·2	1709	81·5	2909	79·4	
Large for gestational age	545	9·5	195	9·3	350	9·5	
Feeding patterns during infancy							0·053
Formula	717	11·8	258	11·7	459	11·8	
Mixed	4359	71·7	1547	70·2	2812	72·5	
Breastfeed only	1006	16·5	398	18·1	608	15·7	
Growth velocity							0·232
Normal growth velocity	2998	61·6	1111	62·7	1887	61·0	
Retarded growth velocity	962	19·8	353	19·9	609	19·7	
Rapid growth velocity	904	18·6	307	17·3	597	19·3	

* *P*-value derived from *χ* ^2^ test between control and intervention.

** Gestational weight gain recommendations provided by the Institute of Medicine (IOM).

Table 2 summarises the findings derived from the univariate models testing the effect of the intervention, perinatal factors, parental socio-demographic and anthropometric characteristics on the changes observed in children’s BMI percentile. The intervention had no significant effect on the change in BMI percentile. Regarding the effect of parental characteristics, parents’/guardians’ underestimation of children’s weight status at baseline was associated with an increase in children’s BMI percentile (1·41, 95 % CI 0·10, 2·72). Furthermore, children with only one or both parents being overweight/obese had a 2·47 (95 % CI 1·66, 3·29) and a 4·00 (95 % CI 2·94, 5·06) increase in BMI percentile, respectively, compared with those children whose parents were under/normal weight. As far as the effect of perinatal factors was concerned, overweight/obesity in mothers before pregnancy was associated with an increase (2·32, 95 % CI 1·41, 3·23) in children’s BMI percentile, while mother’s age at pregnancy was associated with a decrease (–0·10, 95 % CI –0·20, –0·01) in BMI percentile, but only in girls. In addition, children whose mothers exceeded the recommended weight gain during pregnancy and had a higher gestational age significantly increased their BMI percentiles by 1·15 (95 % CI 0·43, 1·86) and 0·25 (95 % CI 0·07, 0·43), respectively. Mixed feeding during infancy was also found to be significantly associated with an increase in BMI percentile (1·86, 95 % CI 0·33, 3·40) compared with formula feeding, but this was observed only in boys. Girls whose mothers smoked during pregnancy significantly increased their BMI percentiles by 1·42 (95 % CI 0·28, 2·56), compared with girls whose mothers did not smoke at pregnancy. Decreases in BMI percentile of –1·26 (95 % CI –2·63, –0·11) and –1·48 (95 % CI –2·59, –0·38) were also observed for those children who were born first and small for gestational age, compared with those born at a rank other than first and appropriate for gestational age, respectively.

**Table 2:** Effect of parental characteristics and perinatal factors on the change in the age- and sex-specific BMI percentile over the 1-year period of the ToyBox intervention – univariate modelling^*^

	Total sample		Boys		Girls	
Factor	Estimate	95 % CI	Estimate	95 % CI	Estimate	95 % CI
Treatment arm						
Control	Reference		Reference		Reference	
Intervention	0·47	−0·37, 1·32	0·97	−0·24, 2·18	0·78	−0·26, 1·83
Parents’/guardian’ perception of child’s weight status						
Correct estimation	Reference		Reference		Reference	
Underestimation	1·41	0·10, 2·72	2·81	0·97, 4·64	1·44	−0·48, 3·37
Parent’s Nationality						
Native	Reference		Reference		Reference	
Non-native parent(s) *v.* Native	0·14	−1·01, 1·30	−0·67	−2·34, 0·95	−0·62	−2·20, 0·95
Parental weight status						
No parent overweight/Obese	Reference		Reference		Reference	
1 parent overweight/obese	2·47	1·66, 3·29	2·98	1·78, 4·18	2·62	1·49, 3·75
Both parents overweight/obese	4·00	2·94, 5·06	3·91	2·36, 5·47	4·40	2·93, 5·87
Parental education						
Mother’s education (<7 years)	Reference		Reference		Reference	
7–12 years	−0·74	−3·97, 2·49	0·55	−4·70, 5·80	−0·61	−4·73, 3·52
13–14 years	−0·98	−4·21, 2·25	−0·16	−5·41, 5·08	−0·92	−5·03, 3·18
15–16 years	−1·18	−4·41, 2·04	−0·24	−5·47, 4·99	−1·16	−5·27, 2·94
More than 16 years	−0·76	−3·97, 2·44	1·01	−4·20, 6·21	−0·72	−4·78, 3·35
Father’s education (<7 years)	Reference		Reference		Reference	
7–12 years	−1·80	−4·54, 0·93	−2·99	−7·22, 1·23	−0·32	−3·94, 3·30
13–14 years	−1·47	−4·21, 1·27	−2·25	−6·49, 1·99	−0·75	−4·38, 2·87
15–16 years	−1·35	−4·10, 1·40	−2·79	−7·05, 1·46	−0·26	−3·90, 3·38
More than 16 years	−0·74	−3·47, 1·98	−1·16	−5·37, 3·05	−0·04	−3·64, 3·56
Maternal pre-pregnancy weight status						
Under/normal weight	Reference		Reference		Reference	
Overweight/Obesity	2·32	1·41, 3·23	2·28	0·95, 3·62	2·36	1·14, 3·59
Maternal age at pregnancy (years)	−0·08	−0·15, 0·00	0·05	−0·05, 0·16	−0·10	−0·20, –0·01
Smoking during pregnancy						
Not smoking	Reference		Reference		Reference	
Smoking	0·20	−0·80, 1·20	2·10	−0·83, 3·36	1·42	0·28, 2·56
Gestational weight gain						
Meet IOM recommended weight gain	Reference		Reference		Reference	
Exceed IOM recommended weight gain	1·15	0·43, 1·86	1·05	0·01, 2·09	0·82	−0·16, 1·80
Gestational age (weeks)	0·25	0·07, 0·43	0·22	−0·05, 0·49	0·15	−0·10, 0·39
Parity						
Other	Reference		Reference		Reference	
First born	−1·26	−2·63, –0·11	−0·60	−2·62, 1·41	−0·29	−2·13, 1·55
Birth weight						
AGA/LGA	Reference		Reference		Reference	
SGA	−1·48	−2·59, –.38	−0·86	−2·50, 0·78	−1·66	−3·16, –0·16
AGA/SGA	Reference		Reference		Reference	
LGA	1·14	−0·01, 2·29	0·73	−1·05, 2·50	0·90	−0·62, 2·41
Breast-feeding						
Formula	Reference		Reference		Reference	
Mixed	0·42	−0·67, 1·51	1·86	0·33, 3·40	1·31	−0·16, 2·75
Breastfeed only	−0·10	−1·43, 1·23	0·63	−1·23, 2·50	0·97	−0·78, 2·72
Growth velocity in the first 6 months						
Normal growth velocity	Reference		Reference		Reference	
Retarded growth velocity	0·52	−0·42, 1·47	0·44	−0·93, 1·81	−0·31	−1·63, 1·01
Rapid growth velocity	−0·12	−1·09, 0·85	0·53	−0·92, 1·98	−0·28	−1·59, 1·03

IOM recommendations = recommendations by the Institute of Medicine (IOM) 2009 report; AGA, normal for gestational age; LGA, large for gestational age; SGA, small for gestational age.

* Estimates from linear mixed effect models with random intercept for country, socio-economic status and school, and fixed effect adjustment for BMI pre-intervention.

The findings derived from the multivariate model that examined the intervention effect and all factors found in the univariate analyses to be significantly associated with the change in BMI percentile are displayed in Table 3. The non-significant effect of the intervention observed at the univariate analysis was also observed at the multivariate model. However, parents’/caregivers’ underestimation of children’s weight status at baseline remained significantly associated with increase in BMI percentile (1·72, 95 % CI 0·22, 3·21). Similarly, children with one (2·34, 95 % CI 1·50, 3·18) or both parents (2·87, 95 % CI 1·61, 4·13) being overweight/obese, as well as children whose mother was overweight/obese before pregnancy (2·03, 95 % CI 0·89, 3·17), were also found to significantly increase their BMI percentile, compared with children with under/normal-weight parents. Girls born small for gestational age showed a decrease in their BMI percentile (–2·75; 95 % CI –4·49, –1·02) compared with their appropriate for gestational age and large for gestational age counterparts. There were no significant interaction effects seen between the intervention and any other factors.

**Table 3:** Effect of parental characteristics and perinatal factors on the change in the age- and sex-specific BMI percentile over the 1-year period of the ToyBox intervention – multivariate modelling*

	Total sample		Boys		Girls	
Factor	Estimate	95 % CI	Estimate	95 % CI	Estimate	95 % CI
Baseline BMI percentile	−0·11	−0·13, –0·10	−0·12	−0·14, –0·09	−0·11	−0·13, –0·09
Treatment arm						
Control	Reference		Reference		Reference	
Intervention	0·34	−0·58, 1·26	0·50	−0·76, 1·77	0·10	−1·01, 1·22
Parents’/guardian’ perception of child’s weight status						
Correct estimation	Reference		Reference			
Underestimation	1·72	0·22, 3·21	2·22	0·15, 4·28	NA	
Parental weight status						
Both underweight/normal	Reference		Reference		Reference	
One parent overweight/obese	2·34	1·50, 3·18	2·54	1·31, 3·76	2·18	1·03, 3·34
Both parents overweight/obese	2·87	1·61, 4·13	2·68	0·83, 4·53	3·27	1·55, 4·99
Maternal pre-pregnancy weight status						
Normal/underweight	Reference		Reference		Reference	
Overweight/obese	2·03	0·89, 3·17	2·05	0·37, 3·73	1·97	0·42, 3·52
Birth weight						
AGA/LGA					Reference	
SGA	NA		NA		−2·75	−4·49, –1·02

AGA, normal for gestational age; LGA, large for gestational age; SGA, small for gestational age.

* Estimates from linear mixed effect models with random intercept for country, socio-economic status and school.

## Discussion

The primary aim of the present study was to investigate whether the ToyBox-intervention affected the change in BMI percentile of preschool children from six European countries, over the course of 1 year. However, the intervention was not found to have any significant effect on the change observed in children’s BMI percentile. Although some of the targeted EBRB, such as prepacked fruit juice consumption^(31)^ and computer/video games use^(32)^, decreased, this favourable impact of the intervention was not followed by a relevant favourable effect on children’s BMI percentile values. The non-significant findings observed in the present study regarding the effect of the intervention on children’s weight trajectory could partly be attributed to the fact that the intervention could not modify all targeted EBRB, such as children’s physical activity levels^(37)^ and TV viewing^(32)^. Parental lack of concern may play a part, as <10 % of children were overweight/obese at baseline and parents of younger children may believe that excessive adipose tissue is typical during development and that overweight/obese children will outgrow their excess body weight^(38)^.

The accuracy of parental perceptions of their child’s weight status could be an important determinant that might have influenced children’s BMI percentile, since parental misperception of their child’s body weight can affect relevant EBRB^(7)^. The percentage of parents who underestimated the weight status of their overweight/obese children was found to be quite high in the present study (70·6 %) and is comparable to relevant percentages reported by a meta-analysis^(6)^ of seventy-eight studies (i.e. 67·5 %; 95 % CI 62·9, 71·7 %, unadjusted). The majority of studies that examine parental misperception of their child’s weight status^(6,7)^ and factors associated with misperceptions^(39)^ have been cross-sectional, while the evidence coming from prospective studies is limited. Regarding the role of parental misperceptions on childhood obesity, it has been hypothesised that correct perception of child’s weight status would help parents realise that they need to promote and model healthier behaviours, as a means to improve the child’s weight trajectory^(6)^. However, this is not confirmed by the results from other studies. An Australian longitudinal study^(40)^ in 3557 children and their parents, reported that children whose parents perceived their 4–5-year-old children as ‘overweight’, gained more weight across the 8-year follow-up than those perceived as ‘normal weight’, while a similar, though non-significant, result was also reported by another study^(41)^. A Dutch study^(42)^ looked at accurate parental perception of child’s body weight at the age of 5 years and subsequent changes in the child’s BMI *z*-score over time. While 85 % of the parents underestimated their overweight child’s weight status, accurate weight status perception was associated with a greater increase in children’s BMI from 5 until 9 years of age, compared with the underestimation of the child’s weight status. In this regard, and despite the fact that the present study showed that underestimation of weight status is associated with an increase of children’s BMI percentile over this important preschool year, previous evidence suggests that parental awareness of the child’s overweight status is not necessarily protective against subsequent increases in body weight. On the contrary, a recent systematic literature review suggested that interventions aiming to correct parental misperceptions of children’s weight status, especially in younger age groups, will most likely not be effective^(43)^.

Although the findings regarding the role of parental perceptions on their child’s weight trajectory are inconsistent, the evidence on the role of parental weight status has been reported to be much stronger. Both paternal and maternal overweight and obesity have been shown to increase the risk of preschool obesity in several countries worldwide, including Greece^(9)^, the UK^(11)^ and Iran^(10)^. Parental overweight and obesity is also an important risk factor for an unfavourable growth trajectory during childhood. In this context, a large study conducted in the UK^(12)^ showed that the increase in the BMI of preschool children between the age of 3 and 5 years was strongly associated with overweight status of either parents. Besides inheritance of genes that confer susceptibility to obesity, parental overweight is also a proxy for shaping the postnatal eating and physical activity environment of children. In most cases, overweight parents create and sustain an ‘obesogenic’ environment (i.e. high-energetic diets and physical inactivity) for themselves and their children^(20)^.

Extensive research that has been conducted during the last decades in the field of the early origins of chronic disease has produced a wide range of evidence and relevant theoretical models that highlight the predisposing role of several perinatal factors on the later manifestation of childhood obesity. These theoretical models suggest that the effect of certain environmental factors during specific, critical periods of early development can lead to permanent physiological and metabolic adaptations that although serve the purpose of improving the chances of fetal and postnatal survival, may become detrimental in the long term and may be expressed at different life stages in the presence of certain environmental influences^(44)^. In this regard, the present study reported mother’s pre-pregnancy overweight/obesity status to be one of the strongest perinatal factors that remained positively associated with children’s BMI percentile change at the multivariate model. Mother’s pre-pregnancy BMI is a well-established risk factor which has been associated with accelerated growth trajectory^(45)^, abdominal adiposity^(17)^ and obesity at preschool years^(20)^. Although gestational weight gain has also been associated with obesity in preschool children^(46)^, the univariate association observed in the present between gestational weight gain above IOM recommendations and increases in boy’s BMI percentile became non-significant after including mother’s pre-pregnancy BMI into the model. This suggests that mother’s pre-pregnancy weight status is a stronger perinatal determinant of children’s weight trajectory at preschool years, a finding that is also supported by a recent meta-analysis which combines a wide range of data from various countries worldwide^(23)^.

The results of the present study derived from the multivariate model also revealed a significant association between size at birth and BMI change at preschool years in girls. More specifically, girls born small for gestational age decreased their BMI percentile compared with girls who were born large for gestational age or appropriate for gestational age. The associations observed between size at birth and adiposity at later life are complex, with the majority of studies however reporting a ‘J’-shaped positive association between birth weight and children’s weight status^(14)^. Regardless of the exact associations, as size at birth is the product of the nutritional and hormonal milieu in which the fetus develops^(44)^, better control of the trajectory of intrauterine growth via the optimisation of maternal nutrition and health status during pregnancy is fundamental.

The present study should be interpreted under the light of its limitations and strengths. The main limitation was that the ToyBox-sample is not fully representative of the source population of preschool children in Europe, due to the sampling of study participants from specific regions in each country. Further limitations include the possibility of recall bias in the assessment of perinatal factors and the reliance on self-reported parental height and body weight data, although this is mitigated by the use of questionnaires with moderate-to-excellent reliability. Regarding strengths, the main study outcome, that is, the change in the child’s BMI percentile, was based on reliable measurements conducted by rigorously trained researchers who achieved excellent intra- and inter-observer reliability^(47)^. Strengths also include the large sample of preschooler children and the cluster-randomised pre- and post-test design including an intervention and control group, as well as the simultaneously consideration of several factors that were previously identified as potential correlates of preschool children’s BMI.

## Conclusion

The present study showed that parental overweight status and parental underestimation of their child’s body weight constitute unfavourable conditions for the BMI trajectory in preschool years. While the ToyBox intervention did not affect BMI percentile change in preschool children, the significant favourable findings observed by ToyBox-intervention implementation, as well as its low resource requirements and use of available personnel and infrastructure, indicate its scalability potential. It is currently expanding in more regions and countries globally, that is, in Argentina, Belgium, Boston, Bulgaria, Ecuador, Estonia, Germany, Greece, Italy, Malaysia, Malta, New Zealand, Nicaragua, Poland, South Africa, Scotland, Spain and UK^(48,49)^. Although not fully supported by the available literature, increasing parental awareness on the health risks associated with overweight might help increase readiness for change, thus positively modifying EBRB that can support a healthier weight status for the entire family. Furthermore, considering the significant positive associations observed between mother’s pre-pregnancy weight status and children’s BMI percentile change at preschool age, any initiatives that can support women to enter pregnancy with a normal body weight but also adopt and maintain a healthy lifestyle for them and their children after birth, could be proved to be effective early preventive measures in tackling childhood obesity.

## Supplementary material

For supplementary material accompanying this paper visit https://doi.org/10.1017/S1368980021001518. click here to view supplementary material
