# Supplementary material for: Prospective BMI changes in preschool children are associated with parental characteristics and body weight perceptions: the ToyBox-study
Source: Public Health Nutr. 2021 Apr 12;25(6):1552–62. doi: 10.1017/S1368980021001518 (PMC9991669; doi:10.1017/S1368980021001518)
Supplement: Supplementary file 1 [file S1368980021001518sup001.docx]

Supplementary Table 1: Interventional impact of the Toybox study on energy related behaviours (ERBRs) and mean process evaluation score (PES) for teachers and guardians.

| **ERBR** | **Interventional impact** | **Time*Group (β)** | **PES _teacher_** | **PES _guardian_** |
| --- | --- | --- | --- | --- |
| Water Consumption^1^ | Pre-packaged fruit juice | -23.5 | 16.3±5.4 /30 | 9.5±5.3 /18 |
| Snacking Behaviour^2^ | None |  | 14.58±3.70 /24 | 8.50±4.39 /17 |
| Sedentary Behaviour^3^ | Computer/video games use – weekday | -3.40 | 15.58 ±5.53 /24 | 8.67±5.50 /17 |
|  | Computer/video games use - weekend | -5.97 |  |  |
| Physical Activity^4^ | None |  | 13.49±2.56 /26 | 6.90±3.50 /17 |

1. Pinket AS, Van Lippevelde W, De Bourdeaudhuij I, et al. Effect and Process Evaluation of a Cluster Randomized Control Trial on Water Intake and Beverage Consumption in Preschoolers from Six European Countries: The ToyBox-Study. PLoS ONE. 2016;11(4):e0152928.
2. De Craemer M, Verbestel V, Verloigne M, Androutsos O, Moreno L, Iotova V, Koletzko B, Socha P, Manios Y, Cardon G. Combining effect and process evaluation on European preschool children’s snacking behavior in a kindergarten-based, family-involved cluster randomized controlled trial: the ToyBox study. International journal of environmental research and public health. 2020 Jan;17(19):7312.
3. Latomme J, Cardon G, De Bourdeaudhuij I, et al. Effect and process evaluation of a kindergarten-based, family-involved intervention with a randomized cluster design on sedentary behaviour in 4- to 6- year old European preschool children: The ToyBox-study. PLoS ONE. 2017;12(4):e0172730.
4. De Craemer M, Verloigne M, De Bourdeaudhuij I, et al. Effect and process evaluation of a kindergarten-based, family-involved cluster randomised controlled trial in six European countries on four- to six-year-old children's steps per day: the ToyBox-study. Int. 2017;14(1):116.

Supplementary Table 2: Included municipalities breakdown by country and socio-economic status.

| **Country** | **Total** | **Low SES** | **Medium SES** | **High SES** |
| --- | --- | --- | --- | --- |
| Belgium | 17 | 5 | 7 | 5 |
| Bulgaria | 34 | 6 | 13 | 15 |
| Germany | 9 | 3 | 3 | 3 |
| Greece | 27 | 11 | 8 | 8 |
| Poland | 15 | 5 | 5 | 5 |
| Spain | 32 | 12 | 12 | 8 |
